# Concordance of maternal and cord blood SARS-COV-2 immunoglobulin seropositivity after COVID-19 infection or vaccination in pregnancy

**DOI:** 10.1177/19345798251315385

**Published:** 2025-02-05

**Authors:** Gladys Rojas, Aarti Jain, Fayez Bany-Mohammed, Philip Felgner, Muhammad Aslam, Cherry C Uy

**Affiliations:** 1 Department of Pediatrics, Division of Neonatology, 12219 University of California Irvine, Orange, CA, USA; 2 Department of Physiology and Biophysics, 197206 University of California Irvine, Orange, CA, USA

**Keywords:** cord blood, COVID-19, immunoglobulin, IgG, IgM, SARS-CoV-2, SARS-CoV-2 vaccine, vertical transmission

## Abstract

**Objective:**

To assess maternal antibody response to Severe Acute Respiratory Syndrome Coronavirus 2 (SARS-CoV-2) infection during pregnancy and subsequent transplacental antibody transfer in cord blood.

**Study Design:**

This is a prospective cohort study of Coronavirus Disease 2019 (COVID-19) polymerase chain reaction (PCR) positive pregnant women and their newborns. SARS-CoV-2 PCR (+) women were enrolled, with SARS-CoV-2 PCR (−) as control. Maternal blood was obtained at enrollment and cord blood collected at delivery. Baseline maternal and infant characteristics and neonatal outcomes were collected. Samples were analyzed using coronavirus antigen microarray containing immunologically significant antigens from SARS-CoV-2 (including nucleocapsid protein [NP], spike protein [S], S1, S2, receptor-binding domain [RBD]) which can detect SARS-CoV-2 immunoglobulin G (IgG) and immunoglobulin M (IgM).

**Results:**

Thirty-seven maternal-cord blood paired samples were analyzed for SARS-CoV-2 IgG or IgM antibodies; 15 out of 20 samples from SARS-CoV-2 PCR (+) and 14 out of 17 from SARS-CoV-2 PCR (−) mothers were IgG positive. 14 out of the 17 SARS-CoV-2 PCR (−) mothers received COVID-19 vaccine during pregnancy. Difference between IgG seropositivity of naturally infected versus vaccinated mothers were significant, 75% versus 100% (*p* = 0.043). IgM antibodies were detected in 10 out of 20 SARS-CoV-2 PCR (+) women but none were detected in cord blood.

**Conclusions:**

Excellent concordance of SARS-CoV-2 IgG antibodies exist between maternal and cord blood. Significantly higher SARS-COV-2 cord blood IgG seropositivity was found in vaccinated versus naturally infected mothers.

## Introduction

COVID-19 caused by severe acute respiratory syndrome coronavirus 2 (SARS-CoV-2) has led to a worldwide pandemic and continues to affect the global population^1^ As of June 2023, the coronavirus pandemic has led to more than six million deaths globally.^2^ The pathogenesis and symptomatology of COVID-19 infection in human ranges from mild or no symptoms to severe respiratory failure, and possibly death.^3^ Elderly individuals and those with pre-existing comorbidities are at greater risk of developing severe symptoms and death.^4^

Although several studies have emerged detailing the course of disease in the adult non- pregnant population, data on the effect of COVID-19 on pregnant women with perinatal infection remains to be limited and continues to be studied.^5,6,7^ The overall risk of COVID- 19 to pregnant women is low, however pregnant women are at higher risk for severe morbidity and mortality.^8,9^ Studies have shown that COVID-19 may be associated with adverse pregnancy outcomes such as increased risks of preeclampsia, preterm birth and others with systemic inflammatory responses, and vascular malperfusion.^6,7,10^ COVID-19 infection during pregnancy has been reported to be significantly associated with increases in some neonatal morbidities including preterm birth.^11,12^ Similarly, data on the neonatal effects of COVID-19 also remains limited.^13,14^ Cases of probable vertical transmission have been published^15^; however, the extent of vertical transmission remains unclear (estimated at 5.7% by one recent review).^16^

Maternal antibodies can be detected in umbilical cord blood as early as the first trimester and are a reliable source of identification of maternal past and recent infections at the time of delivery. They are a key element of neonatal immunity.^17^ It is known that viruses such as *cytomegalovirus* and *herpes simplex virus* can be transmitted to the fetus during primary maternal infection during pregnancy. Antibody response to COVID-19 infection and the extent of placental transfer of antibodies to infants born to women who were infected during pregnancy is critical in understanding potential newborn protection from COVID- 19 disease. When we embarked on our study, data on maternal transfer of anti-SARS- CoV-2 antibodies were somewhat limited.

Our objective was to validate presence of cord blood SARS-CoV-2 IgG and IgM compared to maternal blood antibody and to evaluate immediate newborn outcomes of those infants born to COVID-19 positive women. Since several COVID-19 vaccines became available in the midst of pandemic and were approved for use in pregnant women, we also evaluated the presence SARS-CoV-2 antibodies in cord blood of infants born to mothers who received COVID-19 vaccines.^18,19^ One study found that COVID-19 vaccination during the third trimester of pregnancy resulted in strong maternal IgG response and effective transfer of antibodies from mother to neonate.^20^ Additionally, a small study found that the concentration of antibodies and the antibody response in cord blood correlated with maternal antibody levels following vaccination.^21^

Understanding the dynamics of maternal antibody response to COVID-19 infection during pregnancy and subsequent transplacental antibody transfer is crucial in protecting newborns by providing passive immunity during the first several months of life. We hypothesized that there is a concordance between umbilical cord blood and maternal blood SARS-CoV-2 antibody levels and that in-utero vertical transmission of SARS-CoV-2 can be identified by the presence of cord blood IgM. The primary outcome measure was the presence of SARS-CoV-2 IgG and IgM antibodies in the cord blood of infants born to mothers who were SARS-CoV-2 PCR (+). Immediate neonatal outcomes will be evaluated and will include COVID-19 infection, NICU admission, respiratory complications, length of stay, and neonatal mortality.

## Methods

**Enrollment and Sample Collection:** This is a prospective cohort study conducted from December 2020 to March 2022 at the University of California, Irvine Medical Center and approved by the UC Irvine Institutional Review Board. Eligible pregnant women admitted to labor and delivery (L&D) were identified through screening of electronic medical records. Subjects were recruited and consented in person. Exclusion criteria included failure to obtain consent and inability to obtain cord blood after delivery (placental and cord anomalies).

During the study period, hospital policy required all L&D admissions to be screened for SARS-CoV-2 PCR via nasopharyngeal swab. SARS-CoV-2 PCR (+) and PCR (−) pregnant women were enrolled using written informed consent. Those who were SARS-CoV-2 PCR negative served as control. One (1) mL of blood was collected from consented subject at the time of routine blood draw, and 1 mL of cord blood was collected after delivery. Relevant clinical data from the mother were collected including maternal age, type of delivery, pre-existing comorbidities, birthweight, gestational age at delivery, COVID-19 infection timing, and vaccination status. Neonatal data included gestational age, birthweight, Apgar scores, SARS-CoV-2 PCR status (24 and 48 hours of age), respiratory complications, sepsis evaluation, NICU admission, length of stay, and mortality.

**Data analysis** was performed using JASP software (JASP Team 2023; Version 0.17.3 for Windows 64bit). Continuous variables were analyzed via an independent student t- test (for normally distributed data). Categorical variables were analyzed via Chi-squared test. Ordinal and non-normally distributed variables were analyzed via the Mann-Whitney U test. A *p*-value of < 0.05 was considered significant for all analyses.

**Sample Processing:** Samples were analyzed at the University of California Irvine Felgner laboratory. For this study, COVAM proteome microarrays were custom manufactured from Applied Microarrays (Phoenix, AZ) according to published methods in Felgner laboratory.^22^ The coronavirus antigen microarray has 10 SARS-CoV-2 antigens (including nucleocapsid protein [NP], spike protein [S], S1, S2, receptor-binding domain [RBD]), 3 SARS, 3 MERS, 12 common COV, and 8 influenza antigens. These antigens were provided by Sino Biological U.S. Inc (Wayne, PA). An aliquot of blood samples was taken and centrifuged for 10 minutes at 1000 g to obtain plasma. The plasma was then diluted 1:100 in protein array blocking buffer (Maine Manufacturing, Sanford, ME, USA) to a final concentration of 10 mg/mL, and incubated at room temperature (RT) for 15 min. The Arrays were hydrated in blocking buffer for 10 minutes prior to adding the sera. Arrays were probed with pre incubated serum samples at room temperature for 30 minutes with gentle agitation and were washed three times for 5 minutes each with TBS-0.05% Tween 20 (T-TBS), followed by incubation with custom-made QDOT-conjugated goat anti-human IgG/ IgM/ IgA (Invitrogen, USA) diluted 1:400 in blocking buffer for 1 hour at RT. After incubation in secondary antibodies, arrays were then washed three times with T-TBS, washed with DI water and air dried. For measurement of Fluorescence intensity, ArrayCam 400-S Microarray Imaging System from Grace Bio-Labs (Bend, OR, USA) was used. Spot and background intensities were measured using an annotated grid (.gal) file and captured tiff files were quantified using ScanArray express software (Perkin Elmer, Waltham, MA, USA). The background of array was subtracted from median spot intensity for each antigen and the data was normalized.

**Data pre-processing and normalization:** After importing the raw data into the R programing environment, the signal to noise ratio (SNR) was calculated as the median signal intensity of a given spot divided by the background signal of the vicinity surrounding area. Samples with an average SNR below 2 (calculated for spots with an MFI over 20,000) were flagged for visual inspection or re probing.

Data normalization was performed in two main steps. First, the control spots were normalized using the Quantile Normalization method. Then with the normalized control spots, a rescaling factor was calculated by dividing the sum of the normalized control spots by the sum of the normalized control spots of a reference control sample set (Training Set). For each sample, the spots were then multiplied by this rescaling factor.

**Prediction models:** A set of 200 known COVID-19 Positive and 135 known COVID-19 Negative samples (Training Set) were used to construct the reactivity prediction models. A reactivity cutoff was calculated using a mixture model (using the function “normalmixEM” from the package “mixtools” version 1.2.0). The cutoff was calculated as 3 standard deviations over the mean of the negative curve of the mixture models. For the selection of the serologically significant, or sero-reactive, antigens, Wilcoxon tests for each antigen over the cutoff, was performed, comparing negative group with the positive group and *p* < 0.05 was considered significant.

For the selection of the optimal prediction antigen set, all possible combinations from one to all sero-reactive antigens were used to build a Logistic Regression Model (function “glm” of the “stats” package version 4.0.0). These models were calculated by randomly separating the training set samples into a training and a testing set (at a 70%/30% ratio). For each model a ROC curve was calculated (package pROC version 1.16.2). The curve coordinates were then obtained (function “coords” from the pROC package version 1.16.2). The coordinates were subset to represent specificities of 0.95 or higher and the threshold defined for the highest sensitivity or the Youden Index.

A logistic regression was then calculated using the testing subset and each sample classified as negative or positive by comparison with the threshold. A confusion matrix was then calculated by comparing the predicted outcomes and the known classification (“known negative” or “known positive”) and the prediction specificity and sensitivity stored into a vector. This comparison (from the randomly creation of the training and testing subsets up to the Sensitivity/Specificity calculations) was repeated 1000 times and the average of each antigen combination compared. With the best performing combination of antigens, a final Logistic Regression model was constructed with the full training set and used to classify the test samples.

The blood specimens were tested on the coronavirus antigen microarray (CoVAM) for antibodies that react to different components of the SARS−CoV−2 virus, including IgM antibodies that develop earlier following exposure to a pathogen and IgG antibodies that develop later and persist. The test evaluates the overall reactivity of multiple SARS−CoV−2 antigens and shows IgG and IgM reactivity against individual antigens from SARS−CoV−2 using single antigen logistic regression.

## Results

**Figure 1.**
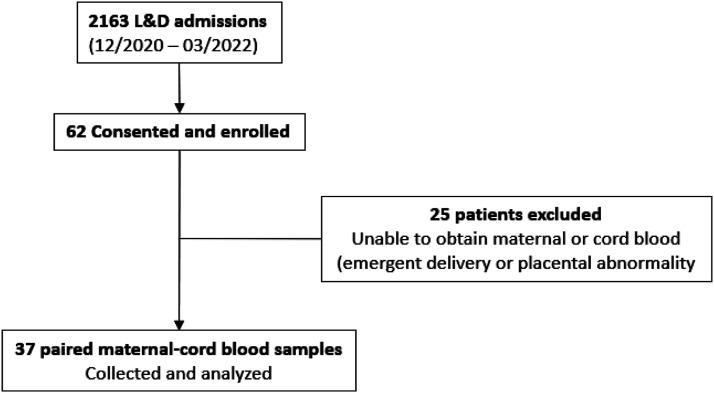
Flow diagram describing patient enrollment.

Sixty-two pregnant women were consented and enrolled. SARS-CoV-2 PCR testing was performed on all 62 women on admission. Twenty-five were excluded secondary to inability to obtain cord blood due to emergent delivery, placental abnormality, or incomplete mother-baby pair. A total of 37 mother-infant paired samples were analyzed (Figure 1). Fifty-four percent (20/37) were SARS-CoV-2 PCR positive, and 46% (17/37) were SARS-CoV-2 PCR negative. SARS-CoV-2 PCR testing was performed on all newborns born to SARS-CoV-2 positive mothers at 24 and 48 hours of age. Maternal and neonatal characteristics were similar between subjects and controls (Table 1). Majority of the subjects were asymptomatic (15 vs 5); out of the five symptomatic women, three presented with mild symptoms (mild fever, cough and rhinorrhea) and two had severe respiratory symptoms requiring ICU admissions. Pre-existing conditions included obesity (19/37), diabetes mellitus (5/27), hypertension (7/37), and hypothyroidism (2/37). Pregnancy complications were observed in few cases such as preeclampsia (5/37), gestational diabetes (4/37), and premature rupture of membranes (3/37). No statistically significant differences were found between the SARS-CoV-2 PCR positive and those were SARS-CoV-2 PCR negative women in all demographic and clinical variables collected (Table 1). Majority of infants were born at >37 weeks gestation (31/37 = 84%), with an average birthweight of 3298 grams. Most neonates had a birth weight >2500 g (92%).

**Table 1.** Maternal demographic characteristics.

	SARS-CoV-2 positive (*N* = 20)	SARS-CoV-2 negative (*N* = 17)
Age	19–36	17–46
Gestational age		
<37 weeks	5	1
>37 weeks	15	16
Delivery		
Vaginal	14	14
Cesarean section	6	3
Comorbidities		
None	2	9
Obesity	10	9
Diabetes mellitus	2	3
Hypertension	4	3
Hypothyroidism	1	1
Other	6	5
Obstetric complications		
Preeclampsia	3	2
Gestational diabetes	2	2
Premature rupture of membranes	2	1
Covid symptoms		
None	15	17
Mild	3	0
Severe	2	0
Vaccinated	0	14

No statistically significant differences in between two gro.

SARS-CoV-2 IgG antibodies were positive in the maternal blood of 15 out of 20 (75%) SARS-CoV-2 PCR positive and in 14 out of 17 (82%) COVID-PCR negative women. Sixty seven percent (10/15) of SARS-CoV-2 PCR positive and IgG positive women were also IgM reactive. Most women who were SARS-CoV-2 PCR (+) on admission were asymptomatic; all symptomatic women were found to be both IgM and IgG reactive. All those who were SARS-CoV-2 PCR (−) but SARS-CoV-2 IgG seropositive (14/17) received COVID-19 vaccine earlier in pregnancy. The difference in IgG seropositivity between naturally infected versus vaccinated women was statistically significant (75% vs 100%, *p* = 0.043).

SARS-CoV-2 IgG antibodies were detected in cord blood of 15 out of 20 (75%) infants born to SARS-CoV-2 PCR positive. However, 14 of 17 (82%) infants born to SARS-CoV-2 PCR negative mothers were also found to have SARS-CoV-2 IgG antibodies in their cord blood; all 14 infants were born to women who were vaccinated against COVID-19. Just like the mothers, the difference in IgG seropositivity between infants born to naturally infected mother and those born to vaccinated was statistically significant, 75% (15/20) versus 100% (14/14), *p* = 0.043.

No IgG seropositive infant was born to an IgG seronegative mother and vice versa. Concordance between maternal and cord blood IgG seropositivity, whether induced by natural infection or vaccination, was 100%. There was no difference between infants born to asymptomatic versus symptomatic mothers when it comes to IgG seropositivity. No SARS-CoV-2 IgM antibodies were detected in cord blood (Table 2).

**Table 2.** Microarray results of maternal and umbilical cord blood.

			SARS-CoV-2 negative *N* = 17	
		SARS-CoV-2 positive *N* = 20	Vaccinated *N* = 14	Unvaccinated *N* = (3)
Maternal blood	IgM reactive	10	0	0
	IgG reactive	15	14	0
Cord blood	IgM reactive	0	0	0
	IgG reactive	15	14	0

*p = 0.043 by Chi-square test (IgG seropositivity in naturally infected mothers and cord blood of their infants vs IgG seropositivity in vaccinated mother and cord blood of their infants).

**Figure 2.**
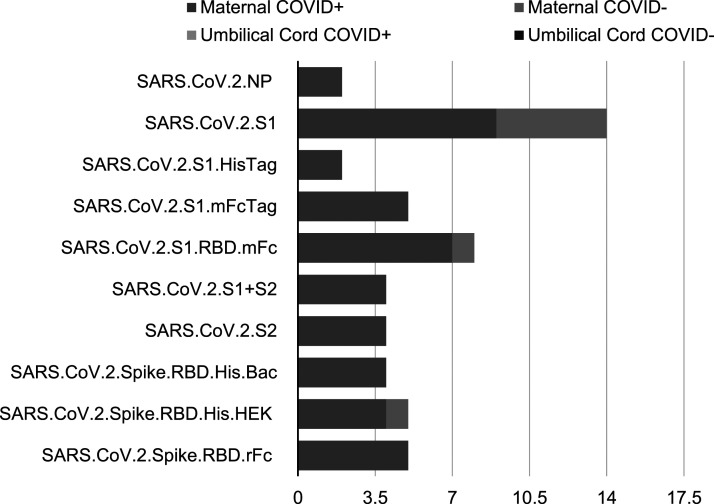
IgM single antigen logistic regression results.

**Figure 3.**
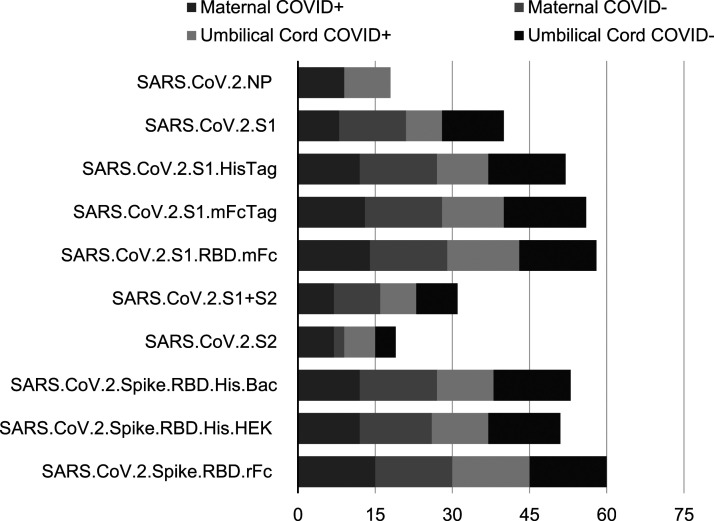
IgG single antigen logistic regression results.

Antibodies were detected using custom microarray that included SARS-CoV-2 specific antigens, such as nucleocapsid and spike proteins (Figures 2 and 3). The majority of IgG antibodies bound to the SARS-CoV-2 spike protein. Among the 20 SARS-CoV-2 PCR positive mothers, 9 (45%) were IgM reactive to the spike protein, while 7 (35%) were IgM reactive to the spike protein’s receptor-binding domain (RBD). All cord blood samples showed no detectable IgM. The majority of SARS-CoV-2 PCR positive mothers were IgG seropositive for both the spoke protein and RBD antigens. Interestingly, cord blood from infants of SARS-CoV-2 PCR negative mothers exhibited similar seropositivity profiles, with IgG reactivity to the spike protein and RBD antigens. Among mothers who tested negative for SARS-CoV-2, 15 out of 17 (88%) were seropositive for the spike protein. Maternal IgM reactivity was more responsive to spike antigens compared to nucleocapsid antigens. Notably, none of the SARS-CoV-2 PCR negative IgG-reactive women tested positive for anti-nucleocapsid antibodies.

Secondary neonatal outcomes were similar among infants born to COVID-19 positive and negative mothers (Table 3). Secondary outcomes in infants born to SARS-CoV-2 PCR positive women were as follows: 5/20 (25%) required NICU admissions, 2/20 (10%) were born prematurely, 1/20 (5%) had low birthweight <2500 grams, 1/20 (5%) underwent sepsis evaluation due to elevated neonatal “sepsis score,” and 2/20 (10%) had mild respiratory symptoms typical of either respiratory distress syndrome seen with premature lungs or retained lung fluid. Length of hospital stay was marginally longer for infants born to COVID-19 positive mothers (3.8 days vs 3.1 days), likely due to 25% of the infants requiring NICU admissions. There were no documented neonatal COVID-19 infection and no neonatal death. All infants were discharged home.

**Table 3.** Neonatal outcomes.

	Infants born to COVID-19 (+) mother (*N* = 20)	Infants born to COVID-19 (−) mother (*N* = 17)
Gestational age		
<37 weeks	5	1
>37 weeks	15	16
Birthweight	3289 g	3307 g
Low birthweight (<2500 g)	2	1
Apgar scores		
1 min	6–9	5–9
5 min	9	9
SARS-CoV-2 PCR positive	0	0
Respiratory complications	2	1
Sepsis	1	1
NICU admission	5	2
Length of stay	3.8 days	3.1 days
Mortality	0	0

No statistically significant differences between the two groups.

## Discussion

Placental transfer of IgG antibodies is an important mechanism that protects the newborn infant from infectious pathogens while the neonatal immune system is still immature and ineffective. We detected SARS-CoV-2 antibodies in the umbilical cord blood of 75% of infants born to women who were SARS-CoV-2 PCR (+) at time of admission to L&D.

This is consistent with published data showing 58%–89% of detected SARS-CoV-2 antibodies in newborns after maternal COVID-19 infection.^19–21,23^ Our study used a novel microarray kit to detect presence or absence of antibodies but did not look at specific titers, therefore we are unable to comment on degree of correlation between maternal and infant antibody levels or the transfer ratio.^22^ The microarray results demonstrated a correlation between maternal and cord blood IgM and IgG antibodies to SARS-CoV-2 spike protein and nucleocapsid antigens.

Vaccination status was determined through chart review and historical data, making it challenging to categorize the timing of vaccination. Based on self-reports, most SARS-CoV-2 PCR negative IgG-reactive vaccinated women received their vaccinations in the second or third trimesters, highlighting the optimal timing for vaccination during the perinatal period to ensure the best immunity. In our study, none of the SARS-CoV-2 PCR positive women had been vaccinated, as the samples were collected before the efficacy of the COVID-19 vaccine was established, resulting in most mothers not having received the vaccine at that time. All infants born to COVID-19 vaccinated mothers had detectable levels of SARS-CoV-2 IgG in their umbilical cord blood. Our data supports that maternal vaccination against COVID-19 leads to a robust and efficient transfer of antibodies across the placenta. Data on placental transmission of antibodies after COVID-19 vaccination has been well documented.

Recently, Sourini et al. showed that vaccine- generated IgG antibodies were detected in all umbilical cord blood samples of their study cohort irrespective of timing and vaccine type given to the mothers.^24^ Similar to our study, Kashani-Ligumsky et al. reported more robust antibody response in women vaccinated during gestation compared to those who contracted the disease during pregnancy.^25^ The same conclusions were reported by Flannery et al.^19^ Given the efficiency of transplacental transfer and higher antibody titers in women who were vaccinated, one can speculate that immunization against SARS-CoV-2 during pregnancy will translate to a better immunologic protection against SARS-CoV-2 infection for the newborn until specific vaccines become available for newborns. Although 50% of SARS-CoV-2 PCR (+) pregnant women in our cohort were SARS-CoV-2 IgM positive, suggesting more recent infection, none was detected in umbilical cord blood, supporting current evidence that SARS-CoV-2 transmission from mother-to-baby is rare.^25,26^

Our data demonstrated excellent concordance between IgG antibodies to SARS-CoV- 2 from maternal and cord blood. Flannery et al. and others demonstrated efficient transfer of maternal IgG antibodies to SARS-CoV-2 across the placenta with transfer ratios increased as time between onset of maternal infection and delivery increases.^19,20,22,27^ Though the duration of passive immunity is limited (up to 6 months), the presence of these antibodies in newborns can be beneficial against COVID-19 infection.^22,27,28^

Published data validate that vaccination against COVID-19 promotes transfer of IgG to the fetus supporting the role of vaccination during pregnancy and in line with data showing COVID-19 vaccination to be effective and safe during pregnancy.^29^ This is important information during counseling of pregnant women who are refusing or are hesitant toward receiving COVID-19 vaccine during pregnancy as it highlights the importance of COVID-19 vaccine for those who are eligible.

The main strength of this study is the use of an innovative, inexpensive, and custom-made microarray which has 10 SARS-CoV-2 antigens (including nucleocapsid protein [NP], spike protein [S], S1, S2, receptor-binding domain [RBD]), 3 SARS, 3 MERS, 12 common COV, and 8 influenza antigens to detect several of SARS-CoV-2 antigens rather than using 1–2 antigen-antibody reactions.^23^ We also used a superior prediction model using logistic regression analysis where each subset sample result was repeated 1000 times and the average of each antigen combination compared thus providing the highest sensitivity and specificity of SARS-CoV-2 antigen and antibody detection. All symptomatic mothers in our study were both IgM and IgG reactive; this finding suggest the high sensitivity of our method to detect COVID-19 infection as IgG and IgM antibodies do not appear until after 1 week from COVID-19 symptom onset and detection happens only toward the middle/end of week two.^30,31^

Our study also supports the current understanding that the risk of in-utero maternal to neonatal transmission is rare as we did not detect any SARS-CoV-2 IgM antibodies in the cord blood of infants born to mothers who were acutely infected at and around the time of delivery and none of the infants tested positive with SARS-CoV-2 PCR at 24 and 48 hours of life.

Limitations of this study include the relatively small sample size conducted at a single institution. Another limitation was that we did not measure antibody titer levels by microarray technique. However, our findings are supported by several recent published data on transplacental transfer of SARS-CoV-2 antibodies. The exact timing of vaccination during pregnancy was not clearly defined. Larger scale studies are necessary to evaluate the transplacental transfer of immunoglobulins based on gestational age and timing during pregnancy.

In conclusion, there is an excellent concordance (100%) between SARS-CoV-2 IgG antibodies in cord and maternal blood of COVID-19 infected or vaccinated mothers and their newborn infants. Cord blood IgG seropositivity was higher in vaccinated mother compared with naturally infected mothers (100% vs 75%). Our study supports the efficient transfer of SARS-CoV-2 maternally derived IgG antibodies. Currently, there is insufficient data in the literature to conclude that maternally acquired SARS-CoV-2 IgG antibodies confers significant immunity to COVID-19 infection during the neonatal period, but they could be crucial in modifying COVID-19 clinical illness or preventing adverse neonatal outcomes.

## Statements and declarations
